# Eco-Stoichiometric Alterations in Paddy Soil Ecosystem Driven by Phosphorus Application

**DOI:** 10.1371/journal.pone.0061141

**Published:** 2013-05-07

**Authors:** Xia Li, Hang Wang, ShaoHua Gan, DaQian Jiang, GuangMing Tian, ZhiJian Zhang

**Affiliations:** 1 College of Environmental and Resource Science, Zhejiang University, Hangzhou, China; 2 Department of Earth and Environmental Engineering, Columbia University, New York, New York, United States of America; 3 China Academy of West Region Development, Zhejiang University, Hangzhou, China; Catalan Institute for Water Research (ICRA), Spain

## Abstract

Agricultural fertilization may change processes of elemental biogeochemical cycles and alter the ecological function. Ecoenzymatic stoichiometric feature plays a critical role in global soil carbon (C) metabolism, driving element cycles, and mediating atmospheric composition in response to agricultural nutrient management. Despite the importance on crop growth, the role of phosphorous (P) in compliance with eco-stoichiometry on soil C and nitrogen (N) sequestration in the paddy field remains poorly understood in the context of climate change. Here, we collected soil samples from a field experiment after 6 years of chemical P application at a gradient of 0 (P-0), 30 (P-30), 60 (P-60), and 90 (P-90) kg ha^−1^ in order to evaluate the role of P on stoichiometric properties in terms of soil chemical, microbial biomass, and eco-enzyme activities as well as greenhouse gas (GHG: CO_2_, N_2_O and CH_4_) emissions. Continuous P input increased soil total organic C and N by 1.3–9.2% and 3%–13%, respectively. P input induced C and N limitations as indicated by the decreased ratio of C:P and N:P in the soil and microbial biomass. A synergistic mechanism among the ecoenzymatic stoichiometry, which regulated the ecological function of microbial C and N acquisition and were stoichiometrically related to P input, stimulated soil C and N sequestration in the paddy field. The lower emissions of N_2_O and CH_4_ under the higher P application (P-60 and P-90) in July and the insignificant difference in N_2_O emission in August compared to P-30; however, continuous P input enhanced CO_2_ fluxes for both samplings. There is a technical conflict for simultaneously regulating three types of GHGs in terms of the eco-stoichiometry mechanism under P fertilization. Thus, it is recommended that the P input in paddy fields not exceed 60 kg ha^−1^ may maximize soil C sequestration, minimize P export, and guarantee grain yields.

## Introduction

The balance of elements has been a main focus of global change ecology and biogeochemical cycling research. Phosphorus (P) application remains an indispensable practice for agricultural crop production. However, P export from soil to surface waters may stimulate outbreaks of water eutrophication [1]. Meanwhile, carbon (C) storage in ecosystems is controlled by the mass conservation principle and the supply of other key nutrients, such as nitrogen (N) and P [2]. Therefore, maintaining a sustainable C-N-P balance in the soil ecosystem is necessary for coping with climate change, maximizing agricultural production, and optimizing P practice.

Ecological stoichiometry (Eco-stoichiometry) is based on stoichiometric theory and the metabolic theory of ecology, which involves the balance of energy and multiple chemical elements in ecological interactions at the subcellular to ecosystem scale [3]. Eco-stoichiometry, expressed as C:N:P stoichiometric ratio, can predict nutrient cycling and microbial biomass production in ecosystems [4], [5], [6] and plays an important role in element regulation during biosphere-scale processes, such as soil C storage and element balance in the soil biomass [7], and also governs greenhouse gas (GHG) emissions in terrestrial ecosystems [8]. Therefore, P fertilization coupled with element eco-stoichiometry may be a determining incentive in defining the dynamics that balance C-N-P and predicting GHG emissions in the soil ecosystem.

Microorganisms drive Earth’s biogeochemical cycles [9] by a “consumer-driven nutrient recycling” (CDNR-like) mechanism that determines nutrient cycling, biomass stoichiometry, and community composition [10], and mediates the global C cycle during climatic changes [11]. In turn, this influences the ecological metabolic rate [4]. Measurements of the proportion of C, N, and P in the microbial biomass may thus be a practical tool for assessing the nutrient limitations of an ecosystem. For example, a low C-to-P ratio of microorganism biomass (MBC:MBP) may stimulate soil microorganisms to release nutrients and enhance the available P pool in the environment, while a high MBC:MBP ratio could cause the microorganisms to compete for available P and enhance soil P immobilization [12]. Conceptually, plasticity and homeostasis are the fundamental mechanisms by which organisms adjust the stoichiometric equilibrium to cope with environmental disturbances [5], [13]. Exogenous P input would alter the primary stoichiometric balances among the soil-microorganisms complex, which could change soil C and N storage. However, the mechanisms on interaction between the exogenous P and soil organism stoichiometry as well as the ecological feedback to dynamics of soil C and N are still unknown.

Eco-enzyme activity represents an intersection of the ecological stoichiometry, wherein eco-enzyme activity (EEA) links environmental nutrient availability with microbial production [3]. Enzyme expression is regulated by environmental signals, while ecoenzymatic activity is determined by environmental interactions [11]. This in turn mediates nutrient cycling, sequestration from soil organic matter, and decomposition biochemistry [3]. The most widely assayed eco-enzymes, β-1,4-glucosidase (BG), β,4-N-acetylglucosaminidase (NAG), leucine aminopeptidase (LAP), and acid (alkaline) phosphatase (AP), hinge functional stoichiometries in relation to organic nutrient acquisition and are used as indicators of microbial nutrient demand [3], [11]. These extracellular enzymes deconstruct plant and microbial cell walls into soluble substrates for microbial assimilation, and are a measure of microbial nutrient demand [14], which reveals the rate limitations of enzymatic catalysis in relation to soil carbon storage [15]. As such, the EEA should be sensitive to the effects of P application on microbial function and provide a mechanistic indicator of P for resource acquisition in the soil ecosystem. However, to date, few studies have focused on microbial function for resource acquisition under P input in soil, and thus the underlying mechanisms are largely unknown.

The interactions among C, N, and P cycling also determine the effect of GHG emissions on the Earth’s climate through their influences on C and N sequestration in soil [16], [17]. Agro-ecosystems contribute a large percentage of global emissions, including ∼60% of N_2_O, ∼39% of CH_4_, and ∼1% of CO_2_ [18]. As one of the important cereal crops, paddy fields (approximately 28.4 M ha) in China contribute to approximately 30% of the total global rough rice yield [17], [19]. Combined with CO_2_, N fertilization may generate intermediate nitrogenous gases (N_2_O) [20]. Better paddy fertilization management strategies are thus needed to mitigate GHG emissions [21] and preserve soil productivity [22]. Relative to N, P is a static entity, which is strongly retained in the soil matrix [23] due to its lack of a significant gaseous phase. Therefore, P management coupled with stoichiometric methodology may offer an advantageous technology for controlling GHGs emitted from paddy fields, but remains inadequately studied.

To date, few investigations have probed the role of P fertilization on soil eco-stoichiometry in paddy fields, which hinders the optimization of C-N-P biogeochemical cycles. In 2005, a paddy field experiment with annual applications of chemical P fertilizer at rates of 0.0, 30, 60, and 90 kg P ha^−1^.y^−1^ was conducted in the Yangtze River delta in southeastern China to understand P driving soil eco-stoichiometry in a paddy field ecosystem. In 2011, we collected soil samples from these experimental paddy plots to investigate the stoichiometry of soil chemical, microbial biomass, and eco-enzyme activities in response to P fertilization. Data on GHG fluxes in July and August were also probed to understand the role of P on typical GHG emissions. We hypothesized that P application could change both soil C-N-P balance and eco-stoichiometric features in paddy soil and thus stimulate soil C sequestration.

## Results

### The Effect of Phosphorus on Soil Biochemical Features and GHG Emissions

**Figure 1:**
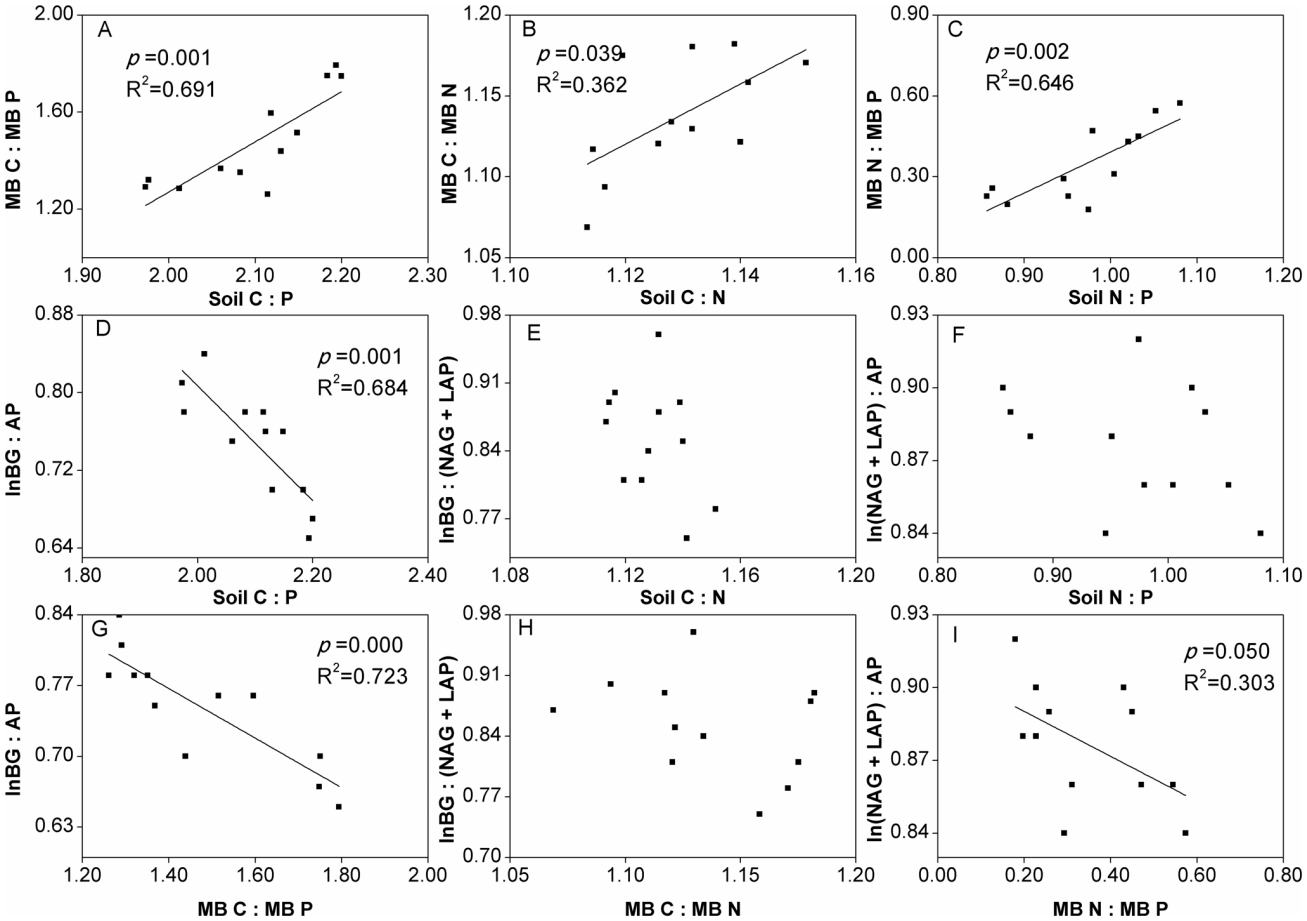
Linear regression of C:N:P stoichiometry for soil chemistry, microbial biomass, and eco-enzymatic activities. Summary of standardized major axis analysis of log10-transformed molar nutrient concentrations. Ratios of C:P, C:N, N:P acquisition activity, as indicated by ratios of ln(BG):ln(AP), ln(BG):ln(NAG+LAP), and ln(NAG+LAP):ln(AP), respectively.

Soils collected from four treatments varied noticeably in chemical, microbial, and eco-enzymatic properties (Table 1). Continuous P application significantly enhanced total soil P by 16–75% compared to P-0. In addition, total soil C was significantly (*p<*0.05) increased by 1.3% to 9.2% for P input compared to P-0, while soil N increased from 3–13% (*p<*0.05). Similarly, soil MBC was increased by 20% to 27% under P-60 and P-90; MBN was also increased under P-60 and P-90 by 28–50%, while an increase in MBP of 52–195% was observed. Due to P application, the ratios of soil MBC:MBP, MBC:MBN, and MBN:MBP were significantly increased with increased ratios of soil C:P (*p*  = 0.001), soil C:N (*p*  = 0.039), and soil N:P (*p*  = 0.002), respectively (Fig. 1). The activities of soil eco-enzymes, such as BG, were enhanced from 39% to 75% compared to P-0, with AP decreasing by 14–33% (*p*<0.01) and NAG+LAP showing no significant changes (Table 1).

**Table 1:** Soil chemical, microbial, and eco-enzymatic properties in the tested paddy field after 5 years of phosphorus application.

Treatment	Soil chemical properties		
	Total C (mmol kg^−1^)	Total N (mmol kg^−1^)	Total P (mmol kg^−1^)
P-0	2070±46^b^	151±3^c^	13.3±0.5^d^
P-30	2096±67^b^	155±5^bc^	15.5±1.0^c^
P-60	2185±91^ab^	162±3^b^	17.9±0.4^b^
P-90	2260±78^a^	171±4^a^	23.3±0.6^a^
**Treatment**	**Soil microbial properties**		
	**MBC (mmol kg^−1^)**	**MBN (mmol kg^−1^)**	**MBP (mmol kg^−1^)**
P-0	40.7±2.3^b^	2.76±0.14^c^	0.83±0.08^d^
P-30	43.9±1.4^b^	3.14±0.22^c^	1.26±0.30^c^
P-60	48.8±2.3^a^	3.54±0.16^b^	2.07±0.22^b^
P-90	51.8±1.9^a^	4.14±0.27^a^	2.45±0.07^a^
**Treatment**	**Eco-enzymatic activity properties**		
	**BG (nmol h^−1^ g ^−1^)**	**NAG+LAP (nmol h^−1^ g ^−1)^**	**AP (nmol h^−1^ g ^−1^)**
P-0	80.0±10.5^c^	276±43^a^	638±36^a^
P-30	111.6±18.0^b^	259±39^a^	576±43^b^
P-60	126.2±8.1^ab^	256±46^a^	538±24^b^
P-90	140.3±15.2^a^	231±35^a^	451±51^c^

The different letters listed beside the data represent significant differences at *p*<0.05 (Duncan test, one-way ANOVA).

**Figure 2:**
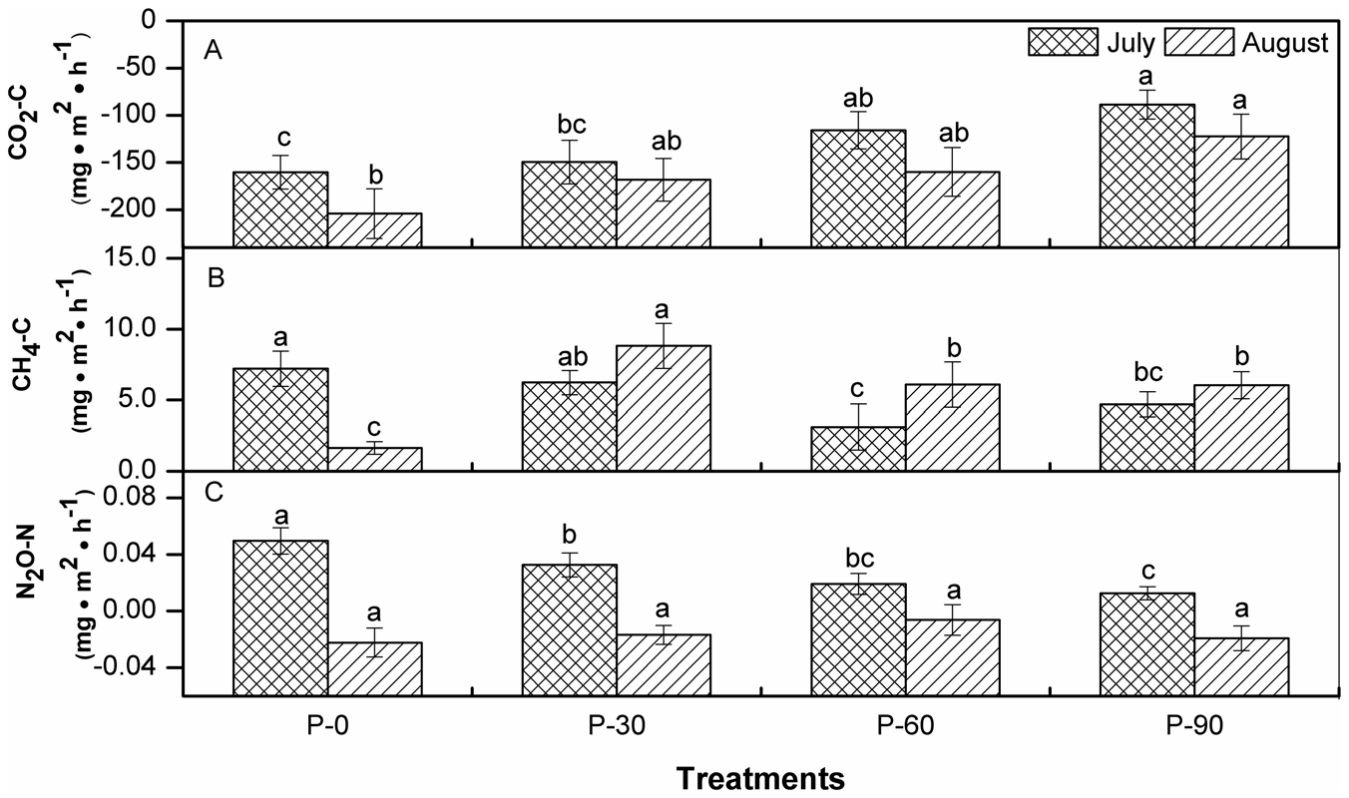
Emission intensities of greenhouse gases responding to phosphorus application for samplings in July and August. The different letters listed above bars represent significant differences at p<0.05 (Duncan LSD test).

Net CO_2_-C flux ranged from −88.7 to −204 mg·m^−2^· h^−1^ and increased significantly (*p<*0.05) with increasing P application (Fig. 2). The net emission of CO_2_ increased by 7–45% under P input compared to P-0 in July, with increases of 17% to 40% in August. CH_4_-C flux, which ranged from 1.7–8.8 mg·m^−2^ h^−1^ in July, was significantly (*p*<0.05) lower by 14%–57% under P input compared to P-0. However, the highest CH_4_ flux occurred with P-30 in August. The N_2_O-N flux in our study ranged from −0.02 to 0.05 mg·m^−2^ h^−1^. N_2_O emission in July was significantly (*p*<0.05) reduced by 34–75% in the tested paddy field with increasing P fertilization compared with P-0; no significant difference was found in August (Fig. 2). Analysis of variance (ANOVA) showed that the soil CO_2_ flux significantly responded to single factors of P application and sampling time; CH_4_ flux significantly responded to single factors of P application and interactions of treatment and sampling date, while N_2_O flux was significantly affected by P application and sampling date as well as interactions (Table 2).

**Table 2:** Results of two-way analysis of variance (repeated ANOVA) showing the *p* values for GHG emissions responding to P application and sampling time (July; August) for paddy field soil.

Factor	CO_2_ flux	CH_4_ flux	N_2_O flux
Treatment	**<0.001**	**0.002**	**0.023**
Time	**0.001**	0.510	**<0.001**
Treatment×Time	0.732	**<0.001**	**0.001**

### The Effect of Phosphorus on Soil Eco-stoichiometry

**Figure 3:**
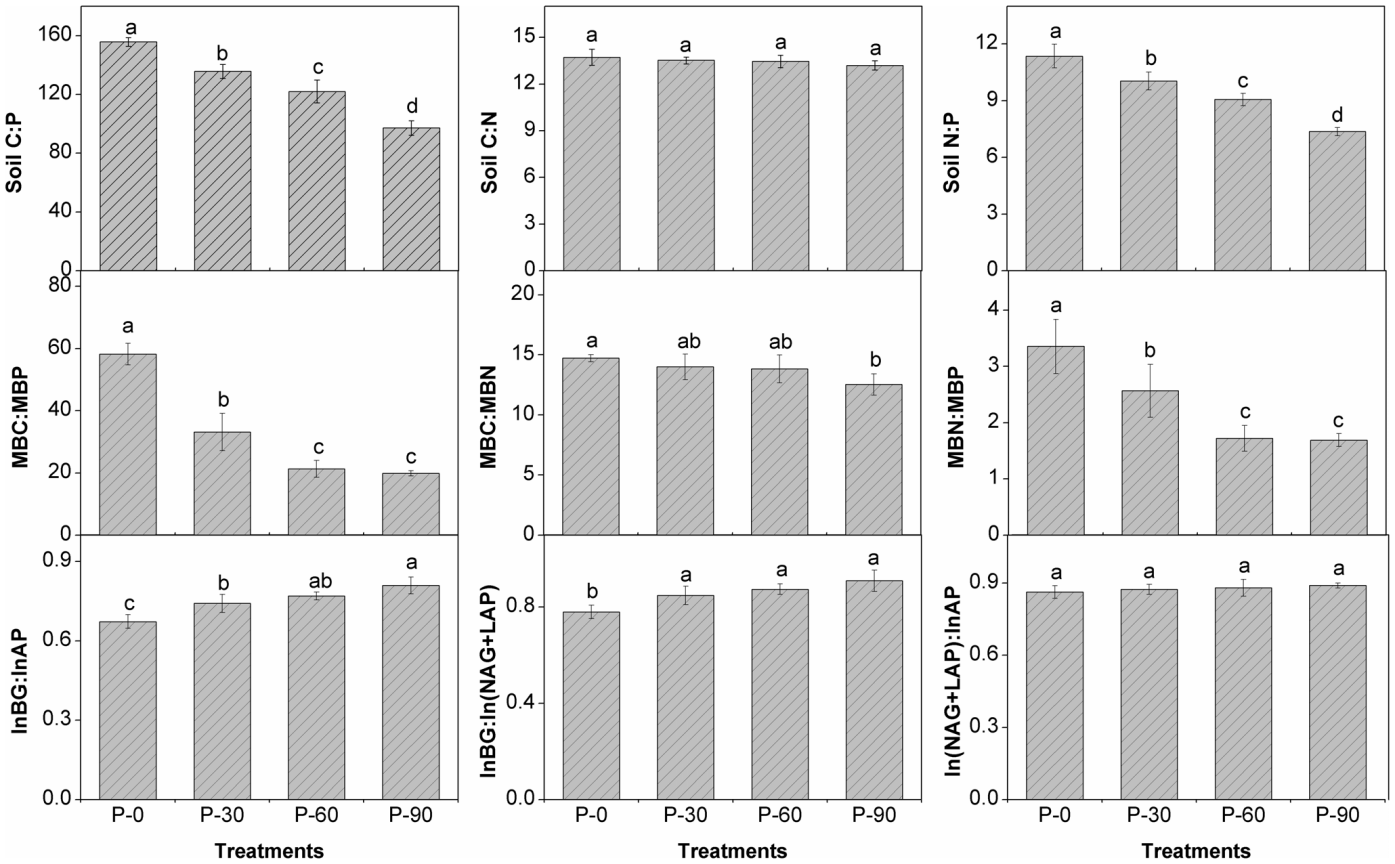
Stoichiometric ratios of soil elements, soil microorganisms, and eco-enzymatic activities in the tested paddy soil under phosphorus treatments. The different letters listed above bars represent significant differences at p<0.05 (Duncan test, one-way ANOVA).

**Figure 4:**
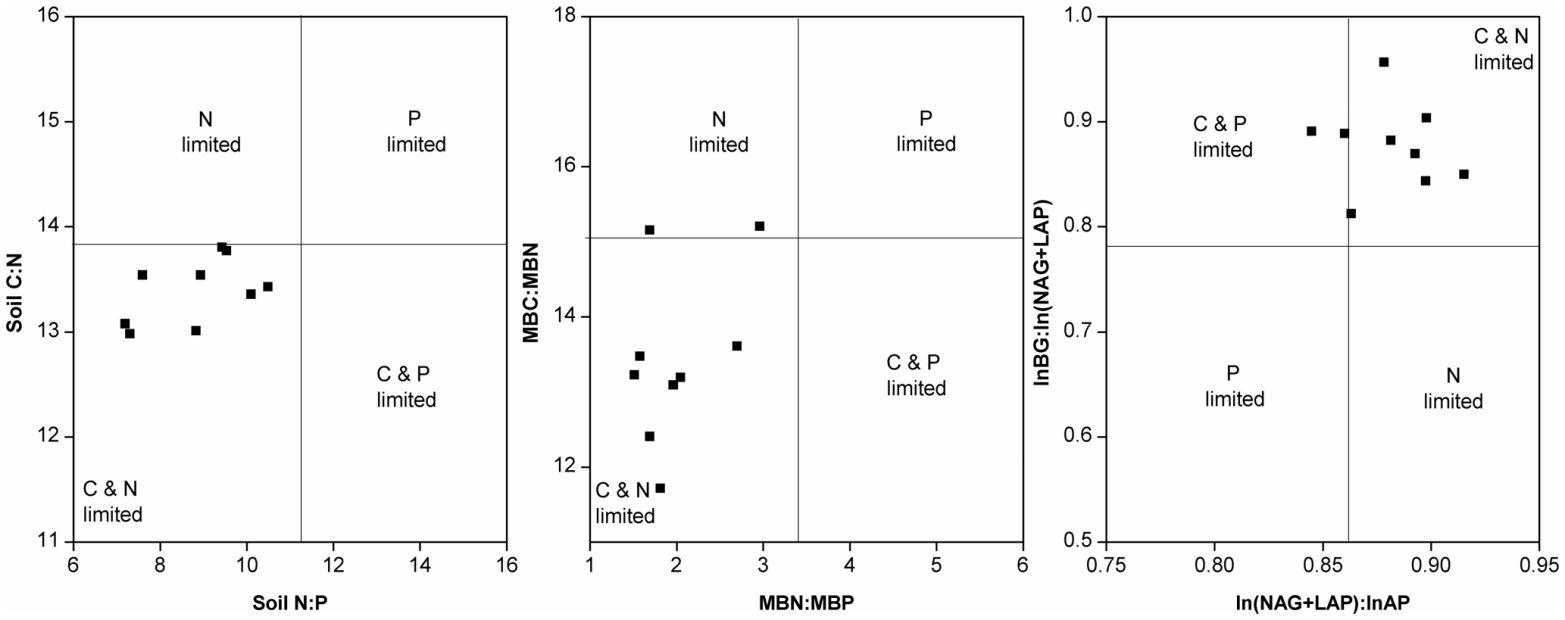
Scatter plots of C:N:P stoichiometry for soil chemistry, microbial biomass, and eco-enzymatic activities for the paddy system under P application compared to P-0.

The stoichiometric ratios of soil C:P declined (from 156 to 97) significantly (*p*<0.01) with increasing P applications, and the ratio of soil N:P was reduced from 11.4 to 7.3 (Fig. 3). However, no significant difference was found for the soil C:N ratio among the four treatments. Soil MBC:MBP and MBN:MBP ratios were both found to be significantly (*p*<0.05) decreased with increases in P application, while the MBC:MBN ratio remained unchanged, except for P-90 (Fig. 3). The acquisition of C relative to organic P indicated by the ratio of ln(BG):ln(AP) was significantly increased with increasing P application (Fig 3). The ln(BG):ln(NAG+LAP), which refers to the acquisition of C relative to organic N, showed no significant differences within P application treatments, but was significantly (*p*<0.01) increased compared to P-0. However, ln(NAG+LAP):ln(AP), which refers to the acquisition of N relative to organic P, showed no significant differences with P application (Fig. 3). Scatter plots of C:N:P stoichiometry for soil chemistry, microbial biomass, and eco-enzymatic activities for the paddy system under P application compared to P-0 indicated that P application caused a C-N co-limitation on ecological stoichiometric processes (Fig. 4). This result suggests that there was a higher C and N demand for soil microorganisms relative to P, integrated soil stoichiometric ratios with biochemical properties, and eco-enzymatic activities (Table 1).

The linear relationships between soil C:P and ln(BG):ln(AP) (*p*  = 0.001), MBC:MBP and ln(BG):ln(AP) (*p*  = 0.000), and MBN:MBP and ln(NAG+LAP):ln(AP) (*p*  = 0.050) were significant and showed a negative trend (Fig. 1). However, there was no significant relationship between soil C:N and ln(BG):ln(NAG+LAP), soil N:P and ln(NAG+LAP):ln(AP), or MBC:MBN and ln(BG):ln(NAG+LAP). The significant relationships showed that soil C:P, soil C:N, soil N:P, soil C:P, MBC:MBP, and MBN:MBP substantially influenced MBC:MBP, MBC:MBN, MBN:MBP, ln(BG):ln(AP), ln(BG):ln(AP), and ln(NAG+LAP):ln(AP), respectively, and could account for the respective 69.1%, 36.2%, 64.6%, 68.4%, 72.3%, and 30.3% variances. Taken together, these results suggest that the microbial biomass stoichiometric ratios were positively influenced by the soil stoichiometric ratios, and both of them were negatively regulated by coenzyme activities under P application.

## Discussion

### Eco-stoichiometry upon Phosphorus Application and the Linkage to Soil Carbon and Nitrogen

Phosphorus amendment increased the decomposition of soil organic C through increased soil respiration [7]. However, the data in Table 1 indicate that paddy soil receiving P application had increased soil C and N pools by 1.3–9.2% and 3%–13%, respectively. Such percent change in soil organic C pools is consistent with a similar long-term field investigation [24], which verified that chemical fertilization (45 kg P ha^−1^) could increase ∼10% higher soil organic C.

The fact that the soil C:P declined (Fig. 1) while soil C and N pools increased (Table 1) upon P input indicated a higher P concentration relative to C in soils, which caused the organic matter pool to become less humic (protein-like) and provided potential energy for microbial utilization [25]. P input modified not only C:N:P ratios in the soil, but also the soil microbial biomass. The positively significant relationships between chemical and microbial stoichiometry (Fig. 1) clearly indicated that microorganisms adapted their acquisition ratios according to local resource ratios. In our study, the decreased soil MBC:MBP upon P input (Fig. 3) demonstrated that P application results in C-limitation in microbial biomass, despite the finding that soil C pools with P input were increased (Table 1). This also suggested that soil microorganisms were limited to soil C allocations and that P enrichment would improve the primary productivity, thus stimulating C sequestration in soil receiving P application, although this C-P synergistic effect [3], [26] would plateau under higher P input rates at P-60 and P-90. Moreover, the distribution of ecoenzymatic C:N:P activity ratios may identify the boundaries of the microbial community response to fluctuations in nutrient availability [3], [11]. The soil microbial metabolic pattern tended to be C limited (Fig. 4) with P input together with the increased ratio of lnBG:lnAP (Fig. 3), which closely responded to the decrease in soil MBC:MBP (Fig. 1). These results indicated that a greater C demand for microbial biomass due to P input enhanced the ecological function of organic C acquisition within the soil-microorganism complex. Hence, our hypothesis that soil C sequestration may be enhanced by P input was verified. Additionally, further analysis showed that MBC:MBN:MBP (49∶3.3∶1.0 to 21∶1.6∶1.0, respectively) with P application (Table 1) deviated significantly from the generally mean atomic C:N:P ratio (60∶7∶1) in the soil microbial biomass [27]. The C:N:P ratio in the biomass reflects the physiological and biochemical constraints on the elemental composition of primary production [6], [27], but differences in soil organism habitats may preclude the emergence of constrained soil microbial element ratios [13]. Our data departed from the mean ratio, which suggested that the overall investment in structural cellular material in the microbial biomass was remarkably affected by P input, indicating that no rigorous homeostasis existed for the soil microbial community in the tested paddy soil.

Soil nutrient enrichment enhances root metabolic activity and the consequent excretion of organic C, which enters the mineral soil through rhizosphere deposition [28] and simulates N fixation by promoting nutrient equilibration [29]. P enrichment would exert a synergistic function of N fixation in the soil-microorganism-plant system [27], which may contribute to soil N accumulation (Table 1), although the soil N:P stoichiometry somewhat decreased in response to P application (Fig. 1). Moreover, the newly-fixed N might be easily stored in the soil and/or biomass under P fertilization, because no significant difference on N-related enzymatic activities was found among the four treatments (Table 1). Based on element stoichiometry [30], [31], soil microorganisms would utilize more plant residues and absorb more C and N for growth with the continuous P application as well as increased soil MBC and MBN (Table 1), which might be favorable for soil C and N sequestration [28], [31] in the paddy ecosystem. However, no significant difference occurred in the lnBG:ln(NAG+LAP) (with the exception of P-0) and ln(NAG+LAP):ln(AP) ratios (Fig. 3) among the P treatments, indicating that a synergistic mechanism among the ecoenzymatic stoichiometry regulates the ecological function of microbial C, N, and P acquisition. Continuous P input caused a significantly (*p<*0.05) negative relationship between the N:P eco-enzymatic and microbial stoichiometries (Fig. 1), which suggests that an abundant source of P in soil microorganisms stimulates the ecological function as a sink for soil N cycle. Together, these results support our hypothesis that P application helps to define the dynamics of the multiple balance of C-N-P in paddy soil, where the synergism of ecoenzymatic stoichiometry enhances soil C and N sequestration.

P application clearly demonstrated a predominance of C and N co-limitations for microorganism metabolic activities and C-N acquisition in our study (Fig. 4). Mechanistically, these may arise through interactions among C, N, and P in bio-molecules such as mRNA [32] and linkage to soil bacterial communities [33], [34]. A biogeochemical equilibrium model verified that ecological stoichiometry constraints microbial community metabolism [11]. Further investigations are needed to determine the inter-annual variations on relationship between molecular biology and eco-enzymatic stoichiometry as well as to illustrate alterations in ecological function caused by long-term P input in paddy ecosystem. It is also an important mechanism that 30–40% of sequestered C would be transferred into sub-soil (below 30 cm) through plant roots in grassland [35]. Although a plow pan layer exists following an arable horizon in a paddy field, the vertical distribution of C sequestration over the entire soil profile at the view of C-P eco-enzymatic stoichiometry is needed to further investigate for paddy plots receiving long-term P application.

### The Role of Phosphorus on Greenhouse Gas Emission

The production of GHG emissions are regulated by soil physical properties and management practices, such as soil temperature, soil O_2_ content, tillage, and alternations of wetting and drying [17], [22], [33]. In our study, the tested paddy soil varied only with P treatments, while the remaining environmental and agricultural factors were kept the same. Therefore, we hypothesized that the shifted GHG fluxes at two typical sampling seasons are mainly related to P treatment. Moreover, because the soil temperature is also a key factor for GHG emissions, July and August were chosen for estimating the GHG emissions subjected to P application due to the higher seasonal temperatures.

The net CO_2_ flux is a balance of photosynthesis and respiration [17]. The negative value of the net CO_2_ flux measured in this study was mainly due to the higher plant CO_2_ uptake, rather than respiration emission. Increases in the soil C pool under continuous P input (Table 1) enhanced CO_2_ fluxes over P application rates for both samplings (Fig. 2). A low P supply (fed by solution culture in 0.5 mg P L^−1^) was found to stimulate root exudation and root aerenchyma development by more than two-fold compared to a high P supply (5–10 mg P L^−1^) [36]. As might be expected, relatively higher CH_4_ emissions were found for P-0 and P-30 sampling in July, while the highest emission occurred for P-30 sampling in August (Fig. 2). Shifting from continuous water-logging to midseason drainage [8], [17], [37] and increases of dissolved oxygen content of the rhizosphere [38], [39] led to a drop in CH_4_ flux and an increase in N_2_O flux in paddy soil. CH_4_ emissions (except P-0) were commonly found to be higher for sampling in July (water-logging) than in August (oxygen secretion through developed roots); however, an overwhelmingly opposite trend occurred for N_2_O for the two samplings. The reason for the latter phenomenon is unclear and will require additional evaluation. The decrease (*p*<0.05) and mostly unchanged N_2_O flux was found upon P application in July and August, respectively (Fig. 2), which is basically consistent with the trend found in the maize system [40]. These results suggest that P input may mitigate N_2_O emission during the high temperature period in this study. Significant relationships between CO_2_ flux and stoichiometric ratios (Table 3) indicated that CO_2_ emission is not only influenced by soil chemical availability, which is basically in agreement with previous reports [7], [17], but is also regulated by the co-limitation of the eco-enzymatic stoichiometric balance for paddy soil receiving P input. Although significant relationships related to stoichiometric ratios were found for both CH_4_ and N_2_O emissions in July, the effect of these stoichiometric ratios for predicting both CH_4_ and N_2_O is completely opposite to that of CO_2_ (Table 3). Moreover, most of these significant relationships disappeared with increasing time (August) after P application. As such, a technical conflict would occur for simultaneously regulating three types of GHGs in terms of an eco-stoichiometry mechanism under conditions of P fertilization, and the associated strategies for such joint regulation would not be synchronous over time. GHG emissions are not only related to agricultural fertilization [38], [39], but also the emission budget and associated net global warming potential, which are closely sensitive to the temporal and spatial traits [17], [20], [37]. Therefore, continuous probing of the eco-stoichiometric mechanisms for yearly GHG budgets responding to P input under the current field survey would be needed.

**Table 3:** Pearson correlation coefficients between CO_2_, CH_4,_ and N_2_O fluxes of paddy field and soil stoichiometric ratios.

Month	GHG flux	Chemical ratio			Microbial ratio			Eco-enzyme ratio		
		C : P	C : N	N : P	MBC: MBP	MBC: MBN	MBN: MBP	ln (BG) : ln (AP)	ln (BG) : ln (NAG+LAP)	ln (NAG+LAP) : ln (AP)
July	CO_2_ flux	−0.871**	−0.578*	−0.851**	−0.732**	−0.547	−0.675*	0.818**	0.786**	0.361
	CH_4_ flux	0.572	0.194	0.589*	0.738**	0.203	0.814**	−0.618*	−0.616*	−0.232
	N_2_O flux	0.841**	0.452	0.839**	0.862**	0.549	0.809**	−0.740**	−0.690*	−0.372
Aug	CO_2_ flux	−0.809**	−0.446	−0.804**	−0.658*	−0.499	−0.555	0.778**	0.822**	0.155
	CH_4_ flux	−0.348	−0.224	−0.341	−0.591*	−0.332	−0.475	0.520	0.414	0.451
	N_2_O flux	−0.195	−0.692*	−0.099	−0.276	−0.216	−0.119	0.093	0.233	−0.308

Soil eco-enzymatic activities are presented as log10-transformed molar nutrient concentrations.

### Implication: Phosphorus Management for Coping with Climate Change

Enhanced soil C sequestration induced by P input (Table 1) is worth considering as a means of addressing climate change. However, such a practice would require technical caution, because the environmental costs of nutrient pollution from agriculture practice have been substantial, including deteriorated downstream water quality and eutrophication [1], [21]. A survey of P application indicated that paddy fields may also act as an ecological sink for P dynamic with no greater than 60 kg ha^−1^ of P input [23], provided that field water management (e.g., water-saving irrigation technology) [41] is optimized and takes into account physicochemical P soil absorption [29], [42]. Supplemental cultivation of green manure during the fallow period (e.g., Chinese milk vetch, *Astragalus sinicus L*) or application of organic fertilizers are well-known means for enhancing soil C and N fixation [34], [43]. Based on the element stoichiometric balance [3], [27] and co-limitation of C and N for soil microbes (Fig. 4) in this study, excessive P in the paddy soil due to continuous P input might be effectively composited with C and N from milk vetch biomass with regard to the synergistic mechanisms among C-N-P in soil-microorganism complexes, leading to further soil C-N sequestration. Currently, rice grain yield (Table S1) among the experimental plots under P-30 (7580 kg ha^−1^) was significantly higher than that of P-0 (6200 kg ha^−1^), while no significant difference was found between P-60 (9100 kg ha^−1^) and P-90 (9070 kg ha^−1^). Grain yield under P-60 is close to the highest level among a survey overview on rice yield [44] in ZheJiang, China. Thereafter, P input to paddy fields at a rate not to exceed 60 kg ha^−1^ is recommended for maximizing soil C sequestration, minimizing P export, and guaranteeing grain yields.

In summary, we found that continuous P application increased the soil C and N pools. P input directly modified soil C:N:P ratios, which shifted the microbial stoichiometry of the biomass to be both C-limited and N-limited. Moreover, a synergistic mechanism among the eco-enzymatic stoichiometry regulates the ecological function of microbial C, N, and P acquisition, thus stimulating C and N sequestration in soil receiving P application in paddy fields. The P application significantly mitigated N_2_O and CH_4_ emissions under P-60 and P-90 compared to P-30 or showed no difference for N_2_O emissions in August, while enhanced CO_2_ fluxes were observed in both July and August samplings. Therefore, these results suggest that technical conflicts would occur for the simultaneous regulation of three major GHGs through an eco-stoichiometry mechanism under the conditions of P fertilization. The integration of soil C sequestration, minimization of P export, and guarantee of the grain yield translate into a recommended P paddy field ecosystem input rate of no greater than 60 kg ha^−1^.

## Materials and Methods

(This work is unrelated to an ethics issues, and no specific permit was required for the described field study.).

### Study Site

A plot experiment on P management for paddy field was established in April 2005 at the demonstration park of YuHang County Agricultural Research Station (30°18′51.84′′N, 119°54′13.37′′E) in ZheJiang, China. The experimental region has a subtropical monsoon climate with an average temperature of 17.8°C and an average annual rainfall of 1450 mm. The soil type is typical clay, blue-purple paddy soil. The dominant soil type in this region is a blue-purple paddy soil (*Mollic Endoaquoll*). The soil before experimental plots construction was composed of 3% sand, 47% silt, and 50% clay in the top 150 mm, while contents of soil total P, total C and total N were found 13.7, 2087, and 148 mmol kg^−1^, respectively. Local farmers in this region routinely apply approximately 25–50 kg P ha^−1^ of inorganic P fertilizer or compound fertilizer in late July or early August in order to support one crop of rice and an over-wintering crop, such as wheat (*Triticum aestivum*) or rape (*Brassica Napus*).

### Rice Field Plot Experiment and Soil and Greenhouse Gases Sampling

#### Rice field plot experiment

The construction of the field experimental plots, including specific designs on plot ridges, trenches, berms and inlets/outlets, was previously described by Zhang [23]. Briefly, twelve 4 m×5 m plots were constructed in two parallel rows in 2005. In order to keep each of these plots hydrologically isolated, a high-density polyethylene impermeable membrane of 0.75 mm (thickness)×105 cm depth was first inserted between two neighboring plots, and then concrete-brick walls of 12 cm (width)×105 cm (depth) were coupled on both sides of the membrane. The experiment was conducted using a completely randomized block design with three replicates for each treatment. P was applied at rates of 0 (P-0), 30 (P-30), 60 (P-60), and 90 (P-90) kg ha^−1^ in June, using superphosphate since 2005. These P treatments cover the routine rate of P application of local farmers and the excessive P rates for field experiments. All plots received 170 kg N ha^−1^ (urea) and 50 kg K ha^−1^ (KCl) each year. In 2011, 25-day-old rice seedlings (*Oryza sativa L*.) were transplanted at 150 mm×150 mm spacing, and rice was harvested on November 10, 2011. Details on rice grain yield and yield components are presented in Text S1.

#### Soil sampling

After application of P for 6 years from 2005 to 2011, the experimental paddy field enters a relatively stable state for soil biosphere. Therefore, soil sampling in 2011 was chosen for a comparison investigation on soil eco-stoichiometric characteristics under different P treatments. On May 21, 2011, one month before rice transplantation, each plot was divided into six subplots of grab-sampling to minimize edge effects. The arable soil samples for each plot consisted of six composited 2 cm diameter×10 cm deep cores (cultivated horizon) [45]. The samples were kept on ice and immediately transported to the laboratory. Air-dried soils were screened through 2 mm sieves and then stored in the dark at 4°C until analysis for total C, N, and P concentrations. The fresh soil samples were stored at 4°C for no longer than one week before microbial C, N, P, and enzyme activity analyses.

#### Gas collection and measurements

Sampling of GHGs was set in July and August 2011 following fertilization, representing the early-phase of rice tilling and middle-phase of rice jointing during the high temperature season. A series of lab-made gas collection apparatuses (Text S2) was constructed prior to field sampling, and then set in the middle of each plot. Gas sampling was performed between 9∶00 am and 11∶00 am using 10 ml vacuum glass tubes connected to the stomata sampling apparatus. For each flux determination, a series of gas samples was taken at 10 min intervals over one hour. At the same time, the air temperature within the chambers were measured, while ambient air pressure was recorded by an automatic weather station (FRT CS01A) assembled 95 m away from the experimental plots. The samples were analyzed for CO_2_, N_2_O, and CH_4_ within 24 h using an Agilent 6890D GC (Agilent Technologies, Palo Alto, California, USA) equipped with an automated gas chromatographic system with an ^63^Ni electron capture detector for N_2_O analyses and flame ionization detector for CO_2_ and CH_4_.

The CO_2_, N_2_O, and CH_4_ fluxes were calculated using linear regression of the change in gas concentration, based on the following equation:
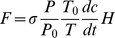
(1)where *F* is the flux rate of CO_2_/CH_4_/N_2_O (mg⋅m^−2^⋅h^−1^), 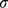
is the CO_2_, CH_4_ and N_2_O gas density in standard state (1.96, 0.71, and 1.96 kg⋅m^−3^, respectively), *P* is the atmospheric pressure (kPa), *T* the air temperature (K), 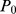 and 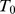
is the atmospheric pressure (101.3 kPa) and the air temperature (273 K), 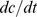 is the unit conversion factors for calculating CO_2_ and N_2_O flux rates, and H is the height of the chamber (m).

#### Soil fraction properties

Air-dried samples were analyzed for total organic C (TOC), total N, and P concentrations according to standard methods described by Bao [46]. The potassium dichromate oxidation method was used to determine total organic C contents. Total N was analyzed by the Kjeldahl method. Total P was digested by HClO_4_-H_2_SO_4_ and measured spectrophotometrically using a continuous flow analyzer (Autoanalyzer III, BRAN+LUEBBE, Germany) set to 880 nm. The fresh soil samples used for determining microbial biomass C and N as well as the P measurements used the fumigation-extraction method as described by Wu et al. [47] and Brookes et al. [48]. Briefly, the fresh soil samples were split into two subsamples with one sample immediately extracted with 0.5 mol L^−1^ K_2_SO_4_ for microbial C and N or 0.5 mol L^−1^ NaHCO_3_ for microbial P, while the other sample was fumigated with chloroform and then extracted. Following centrifugation, C, N, and P concentrations as well as microbial biomass element content were calculated from the difference between the fumigated and non-fumigated soil samples.

### Extracellular Enzyme Assays

Four eco-enzymes (i.e., BG, AP, and NAG and LAP) were selected as indicators of microbial nutrient demand in the cycles of C, N, and P, respectively (Table S2). Fluorescence-based soil assays for β-1, 4-glucosidase (BG), β-1, 4-N-acetylglucosaminidase (NAG), L-leucine aminopeptidase (LAP), and acid phosphate (AP) used MUB-linked and AMC-linked artificial substrates [15], [49] (Table S2). Briefly, Sample suspensions were prepared by homogenizing 1 g (wet weight) of soil with 125 ml of 50 mmol L^−1^ sodium acetate buffer using a vortex shaker for 1 min. The pH of sodium acetate buffer for the soil slurries is 6.0, which is the mean soil pH of the environmental samples [50]. Standard high throughput fluorometric enzyme assays were conducted in 96-well blank fluorescent plates (Corning Inc., costar 3603) after pipetting of buffer, slurries, references, and substrates followed a strict order and position on the well plate according to the order of Saiya-Cork [49] (Table S3). The microplates were covered and incubated in the dark at 20°C for 4 h. Then, 10 µl of 1.0 mol L^−1^ NaOH were added to each well to stop the reaction and increase the fluorescence of the substrates. Different buffers respond to NaOH addition in different ways. Thus, a time frame of 1 min between NaOH addition and the reading of plates in a fluorometer was used to reduce analytical variation [50]. Following the addition of NaOH, fluorescence was measured with a Bio-Tek Synergy HT microplate reader (Bio-Tek Inc., Winooski, USA) with 365 nm excitation and 460 nm emission filters. Enzyme activities were calculated and expressed as nmol h^−1^ g ^−1^.

### Statistical Analysis

A completely randomized design was performed to examine significant differences in soil chemical composition, microbiological properties, eco-enzymes, and their stoichiometry among the various P fertilization treatments (P-0, P-30, P-60, P-90) using two-way analysis of variance (ANOVA) at *p*<0.05 levels. All assays were conducted in triplicate. A principal component analysis (PCA) was used to transform these variables to two factors. The acquisition ratios of ln(BG):ln(AP), ln(BG):ln(NAG+LAP), and ln(NAG+LAP):ln(AP) activities were also calculated and referred to the acquisition of organic C relative to organic P and N as well as organic N relative to organic P, respectively [3]. ANOVA and Pearson correlation statistics were also performed using the SPSS statistical software version 17.0. The clustering method and PCA were performed using the Minitab statistical software version 16.0. The Origin 8.0 (Origin Lab Corporation, USA) was also used for figure preparation.

## Supporting Information

Table S1 Rice grain yield and yield components of experimental paddy field under P fertilization.
(DOC)

Table S2 Extracellular enzymes with corresponding substrate and the corresponding function.
(DOC)

Table S3 The order of pipetting buffer, slurries, references, and substrates in fluorometric enzyme assays.
(DOC)

Text S1 **Rice grain yield and yield components.**
(DOC)

Text S2 **Gas collection apparatus fabrication.**
(DOC)

## References

[1] Sims JT, Simard RR, Joern BC (1998) Phosphorus loss in agricultural drainage: Historical perspective and current research. Journal of Environmental Quality 27: 277–293.

[2] Hobbie SE, Nadelhoffer KJ, Hogberg P (2002) A synthesis: The role of nutrients as constraints on carbon balances in boreal and arctic regions. Plant and Soil 242: 163–170.

[3] Sinsabaugh RL, Hill BH, Shah JJF (2009) Ecoenzymatic stoichiometry of microbial organic nutrient acquisition in soil and sediment. Nature 462: 795–U117. 20010687 10.1038/nature08632

[4] Allen AP, Gillooly JF (2009) Towards an integration of ecological stoichiometry and the metabolic theory of ecology to better understand nutrient cycling. Ecology Letters 12: 369–384. 19379132 10.1111/j.1461-0248.2009.01302.x

[5] Hall EK, Maixner F, Franklin O, Daims H, Richter A, et al (2011) Linking microbial and ecosystem ecology using ecological stoichiometry: A synthesis of conceptual and empirical approaches. Ecosystems 14: 261–273.

[6] Yu Q, Chen QS, Elser JJ, He NP, Wu HH, et al (2010) Linking stoichiometric homoeostasis with ecosystem structure, functioning and stability. Ecology Letters 13: 1390–1399. 20849443 10.1111/j.1461-0248.2010.01532.x

[7] Bradford MA, Fierer N, Reynolds JF (2008) Soil carbon stocks in experimental mesocosms are dependent on the rate of labile carbon, nitrogen and phosphorus inputs to soils. Functional Ecology 22: 964–974.

[8] Zhang Y, Su SL, Zhang F, Shi RH, Gao W (2012) Characterizing spatiotemporal dynamics of methane emissions from rice paddies in northeast China from 1990 to 2010. Plos One 7. 10.1371/journal.pone.0029156 PMC3250406 22235268

[9] Falkowski PG, Fenchel T, Delong EF (2008) The microbial engines that drive Earth's biogeochemical cycles. Science 320: 1034–1039. 18497287 10.1126/science.1153213

[10] Cherif M, Loreau M (2009) When microbes and consumers determine the limiting nutrient of autotrophs: a theoretical analysis. Proceedings of the Royal Society B-Biological Sciences 276: 487–497. 10.1098/rspb.2008.0560 PMC2664333 18854301

[11] Sinsabaugh RL, Shah JJF (2012) Ecoenzymatic stoichiometry and ecological theory. In: Futuyma DJ, editor. Annual Review of Ecology, Evolution, and Systematics, Vol 43. Palo Alto, CA: Annual Reviews. 313–343.

[12] Peng PQ, Zhang WJ, Tong CL (2005) Soil C, N and P contents and their relationships with soil physical properties in wetlands of Dongting Lake fliood plain. Chinese Journal of Applied Ecology 16 (10): 1872–1878. 16422506

[13] Sterner RW, Elser JJ (2002) Ecological Stoichiometry: The Biology of Elements From Molecules to the Biosphere. Princeton, NJ: Princeton University Press.

[14] Moorhead DL, Sinsabaugh RL (2006) A theoretical model of litter decay and microbial interaction. Ecological Monographs 76: 151–174.

[15] Sinsabaugh RL, Lauber CL, Weintraub MN, Ahmed B, Allison SD, et al (2008) Stoichiometry of soil enzyme activity at global scale. Ecology Letters 11: 1252–1264. 18823393 10.1111/j.1461-0248.2008.01245.x

[16] Falkowski P, Scholes RJ, Boyle E, Canadell J, Canfield D, et al (2000) The global carbon cycle: A test of our knowledge of earth as a system. Science 290: 291–296. 11030643 10.1126/science.290.5490.291

[17] Cai ZC (2012) Greenhouse gas budget for terrestrial ecosystems in China. Science China-Earth Sciences 55: 173–182.

[18] OECD (2000) Environmental indicators for agriculture methods and results. Executive summary Paris.

[19] IRRI (2009) World rice statistics: Rough rice production by country and geographical region-FAO1961–2007. Available: http://beta.irri.org/solutions/index.php?option=com_content&task=view&id=250. Accessed 2009 Jul 28.

[20] Davidson EA (2009) The contribution of manure and fertilizer nitrogen to atmospheric nitrous oxide since 1860. Nature Geoscience 2: 659–662.

[21] Vitousek PM, Naylor R, Crews T, David MB, Drinkwater LE, et al (2009) Nutrient imbalances in agricultural development. Science 324: 1519–1520. 19541981 10.1126/science.1170261

[22] Snyder CS, Bruulsema TW, Jensen TL, Fixen PE (2009) Review of greenhouse gas emissions from crop production systems and fertilizer management effects. Agriculture Ecosystems & Environment 133: 247–266.

[23] Zhang ZJ, Zhang JY, He R, Wang ZD, Zhu YM (2007) Phosphorus interception in floodwater of paddy field during the rice-growing season in TaiHu Lake Basin. Environmental Pollution 145: 425–433. 16979805 10.1016/j.envpol.2006.05.031

[24] Huang QR, Hu F, Huang S, Li HX, Yuan YH, et al (2009) Effect of Long-Term Fertilization on Organic Carbon and Nitrogen in a Subtropical Paddy Soil. Pedosphere 19: 727–734.

[25] Marichal R, Mathieu J, Couteaux MM, Mora P, Roy J, et al (2011) Earthworm and microbe response to litter and soils of tropical forest plantations with contrasting C:N:P stoichiometric ratios. Soil Biology & Biochemistry 43: 1528–1535.

[26] Taylor PG, Townsend AR (2010) Stoichiometric control of organic carbon-nitrate relationships from soils to the sea. Nature 464: 1178–1181. 20414306 10.1038/nature08985

[27] Cleveland CC, Liptzin D (2007) C : N : P stoichiometry in soil: is there a “Redfield ratio” for the microbial biomass? Biogeochemistry 85: 235–252.

[28] Ciampitti IA, Garcia FO, Picone LI, Rubio G (2011) Soil carbon and phosphorus pools in field crop rotations in pampean soils of Argentina. Soil Science Society of America Journal 75: 616–625.

[29] Vitousek PM, Porder S, Houlton BZ, Chadwick OA (2010) Terrestrial phosphorus limitation: mechanisms, implications, and nitrogen-phosphorus interactions. Ecological Applications 20: 5–15. 20349827 10.1890/08-0127.1

[30] Hill BH, Elonen CM, Seifert LR, May AA, Tarquinio E (2012) Microbial enzyme stoichiometry and nutrient limitation in US streams and rivers. Ecological Indicators 18: 540–551.

[31] Hessen DO, Agren GI, Anderson TR, Elser JJ, De Ruiter PC (2004) Carbon, sequestration in ecosystems: The role of stoichiometry. Ecology 85: 1179–1192.

[32] Marklein AR, Houlton BZ (2012) Nitrogen inputs accelerate phosphorus cycling rates across a wide variety of terrestrial ecosystems. New Phytologist 193: 696–704. 22122515 10.1111/j.1469-8137.2011.03967.x

[33] Shange RS, Ankumah RO, Ibekwe AM, Zabawa R, Dowd SE (2012) Distinct soil bacterial communities revealed under a diversely managed agroecosystem. Plos One 7. 10.1371/journal.pone.0040338 PMC3402512 22844402

[34] Reganold JP, Andrews PK, Reeve JR, Carpenter-Boggs L, Schadt CW, et al.. (2010) Fruit and soil quality of organic and conventional strawberry agroecosystems. Plos One 5. 10.1371/journal.pone.0012346 PMC2931688 20824185

[35] Bell LW, Sparling B, Tenuta M, Entz MH (2012) Soil profile carbon and nutrient stocks under long-term conventional and organic crop and alfalfa-crop rotations and re-established grassland. Agriculture Ecosystems & Environment 158: 156–163.

[36] Liu DY, Zhang RF, Wu HS, Xu DB, Tang Z, et al (2011) Changes in biochemical and microbiological parameters during the period of rapid composting of dairy manure with rice chaff. Bioresource Technology 102: 9040–9049. 21835612 10.1016/j.biortech.2011.07.052

[37] Shang QY, Yang XX, Gao CM, Wu PP, Liu JJ, et al (2011) Net annual global warming potential and greenhouse gas intensity in Chinese double rice-cropping systems: a 3-year field measurement in long-term fertilizer experiments. Global Change Biology 17: 2196–2210.

[38] Shrestha M, Shrestha PM, Frenzel P, Conrad R (2010) Effect of nitrogen fertilization on methane oxidation, abundance, community structure, and gene expression of methanotrophs in the rice rhizosphere. Isme Journal 4: 1545–1556. 20596069 10.1038/ismej.2010.89

[39] Ma K, Lu YH (2011) Regulation of microbial methane production and oxidation by intermittent drainage in rice field soil. Fems Microbiology Ecology 75: 446–456. 21198683 10.1111/j.1574-6941.2010.01018.x

[40] Adviento-Borbe MAA, Haddix ML, Binder DL, Walters DT, Dobermann A (2007) Soil greenhouse gas fluxes and global warming potential in four high-yielding maize systems. Global Change Biology 13: 1972–1988.

[41] Zhang ZJ, Yao JX, Wang ZD, Xu X, Lin XY, et al (2011) Improving water management practices to reduce nutrient export from rice paddy fields. Environmental Technology 32: 197–209. 21473282 10.1080/09593330.2010.494689

[42] Wang K, Zhang ZJ, Zhu YM, Wang GH, Shi DC, et al (2001) Surface water phosphorus dynamics in rice fields receiving fertiliser and manure phosphorus. Chemosphere 42: 209–214. 11237300 10.1016/s0045-6535(00)00127-2

[43] Lee CH, Do Park K, Jung KY, Ali MA, Lee D, et al (2010) Effect of Chinese milk vetch (Astragalus sinicus L.) as a green manure on rice productivity and methane emission in paddy soil. Agriculture Ecosystems & Environment 138: 343–347.

[44] Wang DY, Zhand XF, Zhou CN, Zheng GS, Zhang GX, et al (2010) Grain yield difference investigation and reasonable planting density analysis of rice production in Zhejing Province. Acta Agricuhurae Zhejiangens 22: 330–336.

[45] Sander T, Gerke HH, Rogasik H (2008) Assessment of Chinese paddy-soil structure using X-ray computed tomography. Geoderma 145: 303–314.

[46] Bao SD (2000) Agro-chemical analysis of soil. Beijing: China Agricultural Press. 78–290.

[47] Wu J, Joergensen RG, Pommerening B, Chaussod R, Brookes PC (1990) Measurement of soil microbial biomass C by fumigation extraction - an automated procedure. Soil Biology & Biochemistry 22: 1167–1169.

[48] Brookes PC, Powlson DS, Jenkinson DS (1982) Measurement of microbial biomass phosphorus in soil. Soil Biology & Biochemistry 14: 319–329.

[49] Saiya-Cork KR, Sinsabaugh RL, Zak DR (2002) The effects of long term nitrogen deposition on extracellular enzyme activity in an Acer saccharum forest soil. Soil Biology & Biochemistry 34: 1309–1315.

[50] German DP, Weintraub MN, Grandy AS, Lauber CL, Rinkes ZL, et al (2011) Optimization of hydrolytic and oxidative enzyme methods for ecosystem studies. Soil Biology & Biochemistry 43: 1387–1397.

