# Correction: Eco-Stoichiometric Alterations in Paddy Soil Ecosystem Driven by Phosphorus Application

**DOI:** 10.1371/annotation/a267ec0a-4ef6-4d9e-821f-5e818dac3d08

**Published:** 2013-12-30

**Authors:** Xia Li, Hang Wang, ShaoHua Gan, DaQian Jiang, GuangMing Tian, ZhiJian Zhang

A funding organization and grant were incorrectly omitted from the Funding Statement. The Funding Statement should read: "The authors wish to thank the National Natural Science Foundation of China (41373073) and Zhejiang Science and Technology Program (2011F20025) for providing the financial support for this project. The funders had no role in study design, data collection and analysis, decision to publish, or preparation of the manuscript. "

