# Supplementary material for: Eco-Stoichiometric Alterations in Paddy Soil Ecosystem Driven by Phosphorus Application
Source: PLoS One. 2013 May 7;8(5):e61141. doi: 10.1371/journal.pone.0061141 (PMC3646879; doi:10.1371/journal.pone.0061141)
Supplement: Table S1 — Rice grain yield and yield components of experimental paddy field under P fertilization. (DOC) [file pone.0061141.s001.doc]

**Table S1 Rice grain yield and yield components of experimental paddy field under P fertilization**

| Treatment | Yield  kg ha^-1^ | 1000-grain weight (g) | Total grains per panicle | Filled grains (%) |
| --- | --- | --- | --- | --- |
| P-0 | 6200±238c | 23.6±2.0b | 136±8c | 77.6±4.5b |
| P-30 | 7582±541b | 25.2±1.1a | 120±10b | 82.8±7.1a |
| P-60 | 9100±608a | 26.3±0.6a | 148±16a | 86.4±5.2a |
| P-90 | 9070±475a | 26.4±1.2a | 122±12b | 85.2±9.2a |
