# Supplementary material for: Eco-Stoichiometric Alterations in Paddy Soil Ecosystem Driven by Phosphorus Application
Source: PLoS One. 2013 May 7;8(5):e61141. doi: 10.1371/journal.pone.0061141 (PMC3646879; doi:10.1371/journal.pone.0061141)
Supplement: Table S2 — Extracellular enzymes with corresponding substrate and the corresponding function. (DOC) [file pone.0061141.s002.doc]

**Table S2** Extracellular enzymes with corresponding substrate and the corresponding function.

| Enzyme | Abbrev | EC* | Substrate | Function |
| --- | --- | --- | --- | --- |
| β-1,4-glucosidase | BG | 3.2.1.21 | 4-MUB-β-D-glucoside | Catalyzes the hydrolysis of glucose from β-D-glucosides, including short chain cellulose oligomers. |
| β-1,4-N-acetylglucosaminidase | NAG | 3.2.1.14 | 4-MUB-N-acetyl-  β-D-glucosaminide | Catalyzes the hydrolysis of glucosamine residues in chitooligosaccharides from chitobiose. |
| L-leucine aminopeptidase | LAP | 3.4.11.1 | L-Leucine-7-AMC | Catalyzes proteolysis of leucine and other amino acid residues as well as methyl esters from the N-terminus of peptides. |
| Acid phosphate | AP | 3.1.3.1 | 4-MUB-phospate | Catalyzes hydrolyses phosphoric under acidic conditions. |

*:EC, enzyme commission number; 4-MUB, 4-methylumbelliferyl; 7-AMC, 7-amido-4-methylcoumarin are cited from literatures

**References:**

1. Sinsabaugh RL, Hill BH, Shah JJF (2009) Ecoenzymatic stoichiometry of microbial organic nutrient acquisition in soil and sediment. Nature 462: 795-U117.

2. Sinsabaugh RL, Lauber CL, Weintraub MN, Ahmed B, Allison SD, et al. (2008) Stoichiometry of soil enzyme activity at global scale. Ecology Letters 11: 1252-1264.
