# Supplementary material for: Eco-Stoichiometric Alterations in Paddy Soil Ecosystem Driven by Phosphorus Application
Source: PLoS One. 2013 May 7;8(5):e61141. doi: 10.1371/journal.pone.0061141 (PMC3646879; doi:10.1371/journal.pone.0061141)
Supplement: Table S3 — The order of pipetting buffer, slurries, references, and substrates in fluorometric enzyme assays. (DOC) [file pone.0061141.s003.doc]

**Table S3** The order of pipetting buffer, slurries, references, and substrates in fluorometric enzyme assays

|  | | Sample suspension  （µl） | Sodium acetate buffer  (µl 50mM) | Fluorescent  Standard  (µl 10µM) | Substrate  Solution  (µl 200mM) | Remarks |
| --- | --- | --- | --- | --- | --- | --- |
| Sample | | 200 | - | - | 50 | Eight replicate wells per sample |
| Controls | Blanks control | 200 | 50 | - | - | Eight replicate wells per sample per plate |
|  | Quench control | 200 | - | 50 | - |  |
|  | Negative control | - | 200 | - | 50 | Eight replicate wells per plate |
| Reference standards | | - | 200 | 50 | - |  |
