# Supplementary material for: Eco-Stoichiometric Alterations in Paddy Soil Ecosystem Driven by Phosphorus Application
Source: PLoS One. 2013 May 7;8(5):e61141. doi: 10.1371/journal.pone.0061141 (PMC3646879; doi:10.1371/journal.pone.0061141)
Supplement: Text S1 — Rice grain yield and yield components. (DOC) [file pone.0061141.s004.doc]

**Text-S1: Rice grain yield and yield components**

At maturity before harvest in 2011, the rice grain yield was measured from 6-m^2^ area sampled within each plot. Yield components were determined from ten hills, which were randomly selected. Panicles were hand-threshed, and filled spikelets were separated from unfilled spikelets by submerging them in tap water. Filled and unfilled spikelets were counted to calculate the number of spikelets per panicle and grain-filling percentage.
