# Supplementary material for: Eco-Stoichiometric Alterations in Paddy Soil Ecosystem Driven by Phosphorus Application
Source: PLoS One. 2013 May 7;8(5):e61141. doi: 10.1371/journal.pone.0061141 (PMC3646879; doi:10.1371/journal.pone.0061141)
Supplement: Text S2 — Gas collection apparatus fabrication. (DOC) [file pone.0061141.s005.doc]

**Text S2 Gas collection apparatus fabrication**

Gas collection apparatuses were constructed prior to field sampling. Briefly, one steel base frame (65 cm × 65 cm × 65 cm) was installed in each pot. The frames were covered with a 2.5 mm thick transparent Plexiglas chamber (60 cm × 60 cm × 60 cm). A water-filled groove of 3 cm depth at the upper edge of the frame ensured an airtight seal. The gas sampling channel was 5.0 mm in diameter and was perforated in the middle of the chamber headspace, while the gas channel (5.0 mm) for flux measurements was perforated on both sides of the chamber at a point below 20 cm. The top surface of the chamber was equipped with a portable gas pump (F6010B12MS, 12V with maximum air flow rate of 40 L min^-1^) and was also linked to gas channels by latex tubing (inner diameter 6.0 mm).
